# Supplementary material for: Study on Rapid Detection of Pesticide Residues in Shanghaiqing Based on Analyzing Near-Infrared Microscopic Images
Source: Sensors (Basel). 2023 Jan 14;23(2):983. doi: 10.3390/s23020983 (PMC9862354; doi:10.3390/s23020983)
Supplement: Supplementary file 1 [file sensors-23-00983-s001.zip › sensors-2092269-supplementary.pdf]

Table S1. Partial pesticide degradation behavior database.

Name of pesticides	MRL in brassica campestris leaves(mg/kg)	Degradation half-life under environmental biological conditions($t_{0.5}$)
Methamidophos	0.05	Loam ±0.583d, Sandy 4.8d
Parathion-methyl	0.02	loam77~154 h
Parathion	0.01	Laboratory soil 3~32d, Field soil 2~58 d, Sandy soil 7 d
Monocrotophos	0.03	Loam 1~5d
Phosphamidon	0.05	Loam 6d, Loam and silt 21~28d, Sandy loam 3d
DDT	0.05	Soil 2~5a
Chlordimeform	0.01	Soil 30d
Fonofos	0.01	Field soil 40d
Cadusafos	0.02	Soil 37.3~42.6d
Coumaphos	0.05	Laboratory sandy loam 300d, Silty soil 200d
Terbufos	0.01	Laboratory soil 5d
Carbofuran	0.02	Field soil 8~10d
Aldicarb	0.03	Laboratory soil 2.4d, Field soil 2d
Isocarbophos	0.05	Field soil 3.3d
Endosulfan	0.05	Field sandy loam α- Endosulfan 7.7d, β- Endosulfan 15.4d
Fipronil	0.02	Field soil 65d
Dicofol	0.01	Laboratory soil 45d
Bifenthrin	0.05	Field soil 95d
Trichlorfon	0.1	Field soil thickness 0~2cm 1d, 2~5cm 7.7d, >5cm 0.5d
Cypermethrin	2	Field soil 2.8d

Table S2. Confusion matrix of two-class classification

Explanation of false positive discussion

		Forecast results	
		With pesticide sprayed	No pesticide sprayed
Actual result	With pesticide sprayed	TP	FN
	No pesticide sprayed	FP	TN

$$ACC = \frac{TP + TN}{TP + TN + FP + FN}$$

$$TPR = \frac{TP}{TP + FN}$$

$$FPR = \frac{FN}{TP + FN}$$

$$TNR = \frac{TN}{FP + TN}$$

$$FNR = \frac{FP}{FP + TN}$$

Table S3. TPR and TNR of best Classification models

Pesticide varieties	Set	Classification method	TPR	FPR	TNR	FNR	Pretreatment method	Parameters used in classification methods

/concentration								
Trichlorfon/ 1g/L	Set 1	KNN	95.65	4.35	86.67	13.33	Standardization or self-scaling	K=3
		Naive Bayes	96.15	3.85	86.79	13.21	Standardization or self-scaling	/
		SVM	97.10	2.9	96.66	3.34	regularization	Linear or RBF kernel function
		BP-ANN	98.55	1.45	96.66	3.34	self-scaling	Times of training=15
Cypermethrin/ 0.1g/L	Set 2	KNN	75.75	24.25	86.67	13.33	Standardization or self-scaling	K=1
		Naive Bayes	70.27	29.73	88.46	11.54	Standardization or self-scaling	/
		SVM	75.75	24.25	86.66	13.34	regularization	RBF kernel function
		BP-ANN	66.66	33.34	93.33	6.67	Standardization /self-scaling	Times of training=15

Table S4. TPR and TNR of best SVM discrimination models based on set 3 and set 4.

Pesticide varieties /concentration	Sample set	Pretreatment method	Nt/Nv	*TPR*（%）	*FPR*（%）	*TNR*（%）	*FNR*（%）
Trichlorfon/ 1g/L	set 3	regularization	263:113	94.93	5.07	91.17	8.83
			225:151	96.66	3.34	81.96	18.04
Cypermethrin/ 0.1g/L	set 4	regularization	189:82	68.42	31.58	68.00	32.00
			162:109	67.69	32.31	68.18	31.82
